# Microbial Shifts in the Intestinal Microbiota of *Salmonella* Infected Chickens in Response to Enrofloxacin

**DOI:** 10.3389/fmicb.2017.01711

**Published:** 2017-09-08

**Authors:** Jun Li, Haihong Hao, Guyue Cheng, Chunbei Liu, Saeed Ahmed, Muhammad A. B. Shabbir, Hafiz I. Hussain, Menghong Dai, Zonghui Yuan

**Affiliations:** ^1^National Reference Laboratory of Veterinary Drug Residues (HZAU) and MOA Key Laboratory for Detection of Veterinary Drug Residues, Huazhong Agricultural University Wuhan, China; ^2^MOA Laboratory for Risk Assessment of Quality and Safety of Livestock and Poultry Products, Huazhong Agricultural University Wuhan, China

**Keywords:** intestinal microbiota, enrofloxacin, 16S rRNA gene, chicken, *Salmonella* Typhimurium

## Abstract

Fluoroquinolones (FQs) are important antibiotics used for treatment of *Salmonella* infection in poultry in many countries. However, oral administration of fluoroquinolones may affect the composition and abundance of a number of bacterial taxa in the chicken intestine. Using 16S rRNA gene sequencing, the microbial shifts in the gut of *Salmonella* infected chickens in response to enrofloxacin treatments at different dosages (0, 0.1, 4, and 100 mg/kg b.w.) were quantitatively evaluated. The results showed that the shedding levels of *Salmonella* were significantly reduced in the high dosage group as demonstrated by both the culturing method and 16S rRNA sequencing method. The average values of diversity indices were higher in the control group than in the three medicated groups. Non-metric multidimensional scaling (NMDS) analysis results showed that the microbial community of high dosage group was clearly separated from the other three groups. In total, 25 genera were significantly enriched (including 6 abundant genera: *Lactococcus*, *Bacillus*, *Burkholderia*, *Pseudomonas*, *Rhizobium*, and *Acinetobacter*) and 23 genera were significantly reduced in the medicated groups than in the control group for the treatment period, but these bacterial taxa recovered to normal levels after therapy withdrawal. Additionally, 5 genera were significantly reduced in both treatment and withdrawal periods (e.g., *Blautia* and *Anaerotruncus*) and 23 genera (e.g., *Enterobacter* and *Clostridium*) were significantly decreased only in the withdrawal period, indicating that these genera might be the potential targets for the fluoroquinolones antimicrobial effects. Specially, *Enterococcus* was significantly reduced under high dosage of enrofloxacin treatment, while significantly enriched in the withdrawal period, which was presumably due to the resistance selection. Predicted microbial functions associated with genetic information processing were significantly decreased in the high dosage group. Overall, enrofloxacin at a dosage of 100 mg/kg b.w. significantly altered the microbial community membership and structure, and microbial functions in the chicken intestine during the medication. This study fully investigates the chicken intestinal microbiota in response to enrofloxacin treatment and identifies potential targets against which the fluoroquinolones may have potent antimicrobial effects. These results provide insights into the effects of the usage of enrofloxacin on chicken and will aid in the prudent and rational use of antibiotics in poultry industry.

## Introduction

*Salmonella enterica* causes significant morbidity and mortality in poultry (Dunkley et al., 2009). Fluoroquinolones (FQs) are important antibiotics used to treat various bacterial infections in chickens in many countries (Martinez et al., 2006; Landoni and Albarellos, 2015). However, high concentrations of FQs achieved in feces after oral administration may result in emergence and dissemination of antimicrobial resistant bacteria and genes (Hooper, 2001; Redgrave et al., 2014). Fluoroquinolones can also have effects on the composition and abundance of a number of bacterial taxa in the chicken intestine (Modi et al., 2014; de Lastours and Fantin, 2015). The antibiotic-resistant bacteria and propagated zoonotic pathogens might be passed to human through contaminated meat and eggs, raising public health concerns (Wang et al., 2006).

The microbial community of the chicken intestine is highly diverse with more than 1000 bacterial species and the population density can reach about 10^11^ cells/g digesta (Stanley et al., 2014; Tang et al., 2014). The enormous and dynamic ecosystem has a symbiotic relationship with its host and assists the host in many physiological processes (Sullivan et al., 2001; Yin et al., 2010; Zhao et al., 2013; Isaac et al., 2017). The balance of microflora is important for reducing pathogen colonization and preventing proliferation of already existing pathogens such as yeasts or *Clostridium difficile* (Sullivan et al., 2001; Looft et al., 2012). Consequently, alterations of the microbiota composition induced by antimicrobials may cause disorder in the ecological balance between microorganisms and host, allow for the proliferation of exogenous pathogens and give rise to serious clinical implications (Kim et al., 2011; Stanley et al., 2014). Thus understanding the effects that specific antimicrobials have on the intestinal microbiota is crucial for clinicians, in order to choose the most efficacious therapeutic options available for treating infections.

Studies of the diversity of microbiota in gastrointestinal tract (GIT) have been promoted by the high throughput sequencing of the 16S rRNA gene. The method allows for an in-depth taxonomic survey of bacterial communities and enhances our capability to understand how antimicrobials alter the ecological communities inhabiting the intestinal tract (Looft and Allen, 2012; Stanley et al., 2013; Mohd Shaufi et al., 2015). Several studies have reported the microbial shifts in animals gut after exposure to antimicrobials using culture independent assessments (Yin et al., 2010; Singh et al., 2012, 2013; Videnska et al., 2013; Looft et al., 2014a,b; Sergeant et al., 2014; Mohd Shaufi et al., 2015). Most of these studies focused on the growth-promotion effect of antimicrobials at subtherapeutic doses, the “dose-effect” relationship between the treatment dosages and the alterations of the intestinal microbiota are not fully understood. Much less is known about the microbial shifts of the pathogens and opportunistic pathogens under the antibiotic pressure that may cause reinfection after withdrawal of the medication.

We are interested in enrofloxacin since it is the most commonly used FQs in poultry in many countries. We performed a clinical efficacy study previously to investigate the effect of enrofloxacin against *Salmonella* infection in chicken. During the experiment, the fecal samples were collected at pre-determined time points and then subjected to the 16S rRNA sequencing. Since healthy animals would not be medicated in typical clinical efficacy trials, non-*Salmonella* infection groups were not included in the present study. The effects of enrofloxacin were evaluated at three dosages. High dosage of 100 mg/kg body weight (b.w.) is the highest non-toxic dosage of enrofloxacin in chicken (Maslanka et al., 2009). The middle dosage of 4 mg/kg b.w. is designed based on our previous pharmacokinetics/pharmacodynamics (PK/PD) study of enrofloxacin against *Salmonella*. The low dosage (0.1 mg/kg b.w.) is a carry-over dosage which might result from contamination of feed with antibiotics, caused by carry-over of medicated feed to regular feed, or by left-over quantities in the drinking-water system (Scherz et al., 2014). According to the manufacturer label directions of enrofloxacin (Baytril 10% oral solution), for the treatment of Salmonellosis in chicken, enrofloxacin should be administered for 5–10 days. Thus, the treatment duration was set at 7 days to investigate the therapeutic outcome of enrofloxacin against *Salmonella* infection in chicken. The 7 days withdrawal period was designed to detect the microbial shifts in the absence of antibiotic selective pressure (Landoni and Albarellos, 2015). Three rounds of treatments were implemented since antimicrobials may be repeatedly used due to re-infection after withdrawal of the medication. In this paper, we sought to characterize the microbial shifts in the intestinal microbiota of *Salmonella* infected chickens in response to different dosages of enrofloxacin and the changes of microbial composition after withdrawal of antimicrobials.

## Materials and Methods

### Chicken and Sample Collection

Protocols involving animals in this study were carried out in accordance with the recommendations of the committee on the use and care of the laboratory animals in Hubei province and approved by the Animal Care Center, Hubei Science and Technology Agency in China (HZAUCH-2015-008).

**FIGURE 1:**
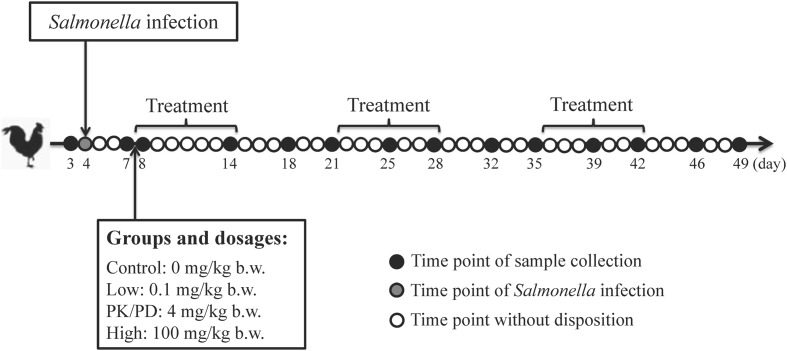
Animal experiment design. Twenty SPF chickens were experimentally infected with *Salmonella* Typhimurium CVCC541 at 4 days old and allocated into four groups of five each at 7 days old. Each group was orally administered with enrofloxacin at dosages of 0, 0.1, 4, and 100 mg/kg b.w. for three rounds of 7-day treatment following with 7-day withdrawal. Clocal swabs were collected from each chicken at pre-determined time points.

Twenty specific-pathogen-free (SPF) male chicks (1-day-old) were randomly separated into four groups of five each and each group was reared in an individual isolator. All the chickens received non-medicated feed and water *ad libitum* and were monitored for conditions twice daily throughout the experiment. At 4 days old, all the chicks were infected with a standardized inoculum of ∼10^8^ CFU per bird of *Salmonella* Typhimurium CVCC541 by gastric gavage. At 8 days old, chickens were orally administered with different dosages of enrofloxacin (Baytril 10% oral solution) (group A: control group, group B: low dosage of 0.1 mg/kg b.w., group C: PK/PD dosage of 4 mg/kg b.w. (based on a previous PK/PD study) and group D: high dosage of 100 mg/kg b.w.). The treatment protocol was three rounds of 7-day treatment following with 7-day withdrawal. Cloacal swabs were collect from each individual chicken at ages of 3, 7, 8, 14, 18, 21, 25, 28, 32, 35, 39, 42, 46, and 49 days and transferred to a sterile 10 mL centrifuge tube (**Figure 1**). All the 280 fecal samples were stored at -80°C untill further analysis.

### *Salmonella* Viable Cell Populations

Since *Salmonella* Typhimurium infection induced acute outbreaks exhibiting clinical disease mainly in chicks younger than 2 weeks old, infection was self-limiting and resulted in asymptomatic intestinal infections in elder chickens, so the shedding levels of *Salmonella* was determined by culturing method. From each cloacal sample, 0.2 g of the mixed feces was taken and emulsified in 1 mL of sterile saline, decimal dilutions were then steaked onto CHROMagar Salmonella agar plates (CHROMagar, France). *Salmonella* appearing typical purple colonies were enumerated after incubation at 37°C for 24 h.

### DNA Extraction, 16S rRNA Gene Amplification and Sequencing

Since a large number of fecal samples (*n* = 280) were collected during the whole experiment and it was reported that animal-to-animal variation could be minimal in genetically similar production animals (Kim et al., 2011), fecal samples collected from the five chickens in the same group at each sampling time point were pooled for DNA preparation and sequencing, neglecting the variations of microbiota between individual chickens, and focusing on the variations induced by antibiotic therapy at different dosage levels and the differences between treatment and withdrawal period.

Total DNA of microbial genomic was extracted from 0.25 g aliquots of each fecal sample using QIAgen DNA Mini Stool kit (QIAGEN) by following manufacturer’s guidelines. The concentration and quality of extracted DNA were confirmed by a NanoDrop spectrophotometer and agarose gel electrophoresis respectively. Extracted DNA was stored at -20°C until further analysis.

Amplification of the V4 region of bacterial 16S rRNA was conducted with the barcoded fusion primers (forward primer: 520F: 5-AYTGGGYDTAAAGNG-3 and reverse primer 802R:5-TACNVGGGTATCTAATCC-3) (Niu et al., 2015). Amplification was performed by using the following cycling conditions: 98°C for 3 min and 25 cycles of 98°C for 30 s, 50°C for 30 s and 72°C for 30 s, followed by 72°C for 5 min. PCR products were separated by a 1.5% agarose gel electrophoresis. DNA was extracted from the gel and then purified by QIAquick Gel Extraction Kit (QIAGEN). The PCR products from all the 20 chickens at ages of 3 and 7 days were pooled, representing samples of pre-infection and pre-treatment, respectively. For the 12 remaining time points, PCR products from the five chickens in the same group were pooled in equimolar ratios. Amplicons of the barcoded V4 region were sequenced using IlluminaMiSeq and the paired-end method with a 7-cycle index read. The sequencing results were denoised using the mothur software package, by following Miseq analysis pipeline SOP (Kozich et al., 2013). Then the sequences were filtered and screened to improve quality, only sequences with an overlap longer than 10 bp and without any mismatch were assembled. Barcode and sequencing primers were trimmed from the assembled sequence. Trimmed and assembled sequences were uploaded to QIIME for further analysis (Niu et al., 2015).

### Data Analysis

Taxonomy abundance was classified by aligning the sequences from each sample to the SILVA bacteria reference database (Quast et al., 2013) using the best hit classification option in Quantitative Insights Into Microbial Ecology (QIIME). The uclust function in mothur was used to generate the bacteria operation taxonomic units (OTU) (97% similarity cutoff) with normalized data (Zhao et al., 2015). All the samples were analyzed by treatment and dosage. Fecal samples collected within the three treatment periods (day 8, 14, 25, 28, 39, and 42) were considered as repeats for treatment. Fecal samples collected within the three withdrawal periods (day 18, 21, 32, 35, 46, and 49) were considered as repeats for withdrawal. The core and unique OTUs between the treatments and withdrawals in each group, among the treatments of the four groups and among the withdrawals of the four groups were compared by Venn diagram. Non-metric multidimensional scaling (NMDS) based on UniFrac distance matrices was performed to visualize the clustering of microbial community of the four groups. OTU accumulation curves and community metrics (inverse Simpson, Shannon, Chao 1) were generated using mothur. Multiple *t*-tests with Holm-Sidak correction were used to detect the statistical significance of alpha diversity indexes between the treatments and withdrawals in each group. The statistical variations among the treatments of the four groups and among the withdrawals of the four groups were examined by one-way analysis of variance (ANOVA) with Bonferroni’s test using Graphpad Prism 6.02. Metastats statistical software was used to identify differentially abundant phyla and genera between the treatments and withdrawals in each group, the treatments of each medicated group with the control group, and the withdrawals of each medicated group with the control group. Analysis of similarities (ANOSIM) was also performed in mothur to test the significant differences between groups.

Microbial functions were predicted using Phylogenetic Investigation of Communities by Reconstruction of Unobserved States (PICRUSt) (Langille et al., 2013; Casaburi et al., 2016). The predicted functions of these genes were aligned to Kyoto Encyclopedia of Genes and Genomes (KEGG) database. STAMP software was used to test the differences among the four treatment groups (Parks and Beiko, 2010; Zhao et al., 2015). ANOVA with Bonferroni’s test were used for multiple-group analysis.

### Data Presentation

Differences were accepted as significant for *p*-values < 0.05. Data are deposited in NCBI’s Short Read Archive (SRA) under accession numbers SAMN06670695- SAMN06670744 and are associated with Bioproject PRJNA381125.

## Results

### Shedding Levels of *Salmonella* in Response to Enrofloxacin Treatment

**FIGURE 2:**
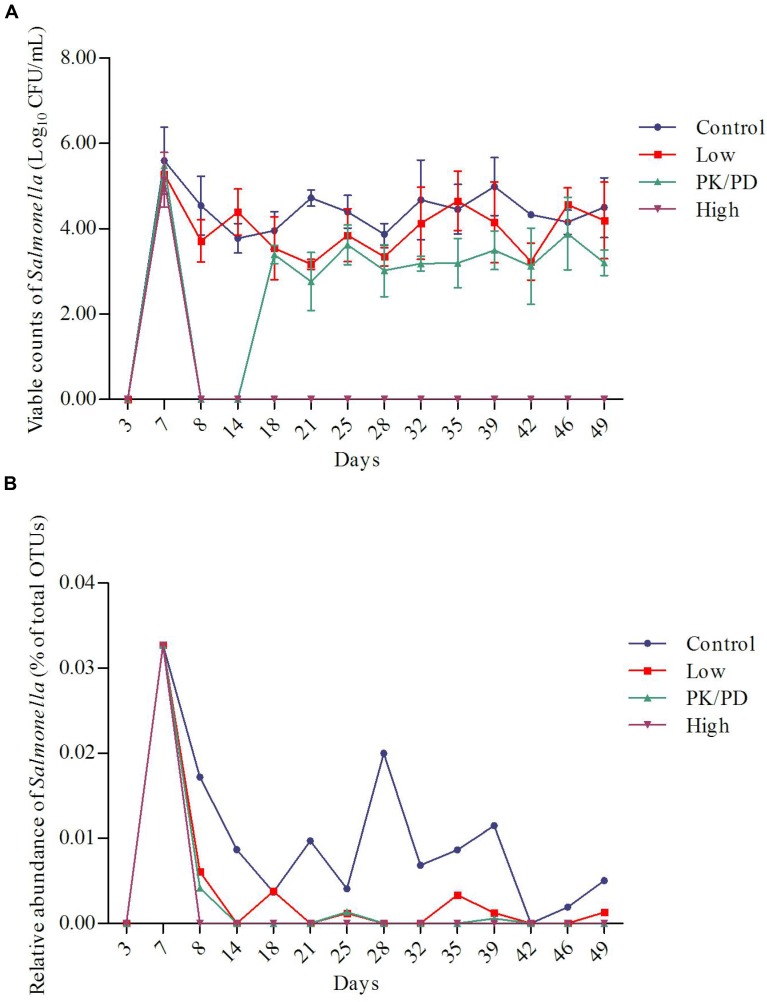
Plots of the changes of *Salmonella* along with time in the four groups determined by culturing method **(A)** (*n* = 5) and 16S rRNA gene sequencing method **(B)**.

By using the culturing method, the shedding levels of *Salmonella* from chicken’s GI tract were determined. The shedding levels reached on average of 6.3 × 10^5^ CFU/g feces per bird post infection. In the control group and low dosage group, the shedding levels remained relatively stable during the whole experiment. In the PK/PD dosage group, the *Salmonella* was inhibited during the first 7-day treatment period, but recovered in the withdrawal period. High dosage of enrofloxacin virtually eradicated *Salmonella* from chicken’s GI tract (**Figure 2A**). Significant differences were observed between each two groups except between the control group and the low dosage group (**Table 1**).

**Table 1:** Significant differences of the viable counts of *Salmonella* (determined by culturing method) and relative abundance of *Salmonella* (determined by 16S rRNA gene sequencing method) among the four groups.

	Viable counts of *Salmonella* (Log_10_ CFU/mL)		Relative abundance of *Salmonella* (% of total OTUs)	
		
Dosage group	Treatment^1^	Withdrawal^2^	Treatment^1^	Withdrawal^2^
Control	4.32^a^	4.41^a^	0.0102^a^	0.0051^a^
Low	3.78^a^	4.04^a^	0.0014^b^	0.0014^b^
PK/PD	2.21^b^	3.27^b^	0.001^b^	0^b^
High	0^c^	0^c^	0^b^	0^b^


*^a-c^Mean values bearing different superscript letters across a column are significantly different (*p* < 0.05). ^1^Fecal samples collected within the three treatment periods (day 8, 14, 25, 28, 39, and 42) were considered as repeats for treatment. ^2^Fecal samples collected within the three withdrawal periods (day 18, 21, 32, 35, 46, and 49) were considered as repeats for withdrawal.*

The changes of relative abundances of genus *Salmonella* determined by 16S rRNA gene sequencing are illustrated in **Figure 2B**. The proportions of *Salmonella* increased from 0% (day 3: pre-challenge) to 0.033% (day 7: post-treatment) per bird. In the control group, the proportions of *Salmonella* decreased as the chicken aged. High dosage of enrofloxacin completely eradicated *Salmonella* immediately after the treatment started (data not shown). The *Salmonella* was significantly reduced to a lower level in the low dosage group (0–0.006%) and PK/PD dosage group (0–0.004%). Significant differences of the treatments were observed between each medicated group and the control group (**Table 1**).

### DNA Sequence Data and Quality Control

In total, 5,250,232 DNA sequence reads were generated from the 50 fecal samples. Over 96% of the sequence reads passed quality control. The average number of sequence reads per sample was 100,894, with a minimum number of 20855 and a maximum number of 193,719. The median sequence read length was 237–238 bases with no ambiguous bases. A total of 17590 OTUs were identified using a sequence identity cutoff of 97%. Rare OTUs (less than 0.001% of the total sequences) were removed to reduce the noise. After this modification step, a total of 1428 OTUs were identified as abundant OTUs and used for further analysis.

**FIGURE 3:**
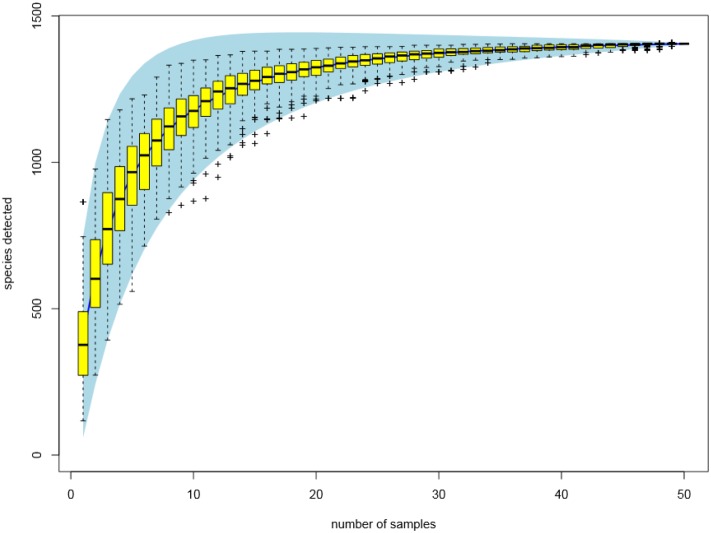
Rarefaction curves of all the fecal samples (*n* = 50) clustered at 97% sequences identity.

The rarefaction curves (**Figure 3**) showed that the sampling had reached saturation since high sampling coverage (∼99%) was achieved for all the samples. The alpha diversity of microbial communities was measured using indices of Chao1, inverse Simpson and Shannon. The Chao1 index reflects how many different taxa are present in the sample (richness). While inverse Simpson and Shannon indices represent how much is the difference among the abundance of different taxa (diversity). The average values of Chao1 were highest in the high dosage group, and followed by control group, PK/PD group and low dosage group (**Table 2**). The average values of microbial diversity indices of the three medicated groups were consistently lower than the control group. No significant differences were observed for the three indices between the treatments and withdrawals in each group, among the treatments and withdrawals of the four groups.

**Table 2:** Community metrics (Chao 1, Simpson and Shannon indices) of the four groups.

	Richness index			Diversity indices					
		
	Chao 1			Simpson			Shannon		
			
Dosage group	TT^1^	WD^2^	Total^3^	TT^1^	WD^2^	Total^3^	TT^1^	WD^2^	Mean^3^
Control	278.17	182.67	230.42	0.71	0.67	0.69	3.41	3.05	3.23
Low	171.67	123.83	147.75	0.60	0.50	0.55	2.54	1.80	2.17
PK/PD	178.67	194.83	186.75	0.51	0.74	0.63	2.19	3.16	2.68
High	265.17	240.17	240.17	0.68	0.55	0.62	3.46	2.40	2.93


*TT, treatment; WD, withdrawal. ^1^Fecal samples collected within the three treatment periods (day 8, 14, 25, 28, 39, and 42) were considered as repeats for treatment. Values are means (*n* = 6). ^2^Fecal samples collected within the three withdrawal periods (day 18, 21, 32, 35, 46, and 49) were considered as repeats for withdrawal. Values are means (*n* = 6). ^3^Mean values of the community metrics calculated from the total 12 samples collected post medication in each group. Values are means (*n* = 12).*

### Microbial Composition and Structure Changed in Response to Enrofloxacin Treatment

**FIGURE 4:**
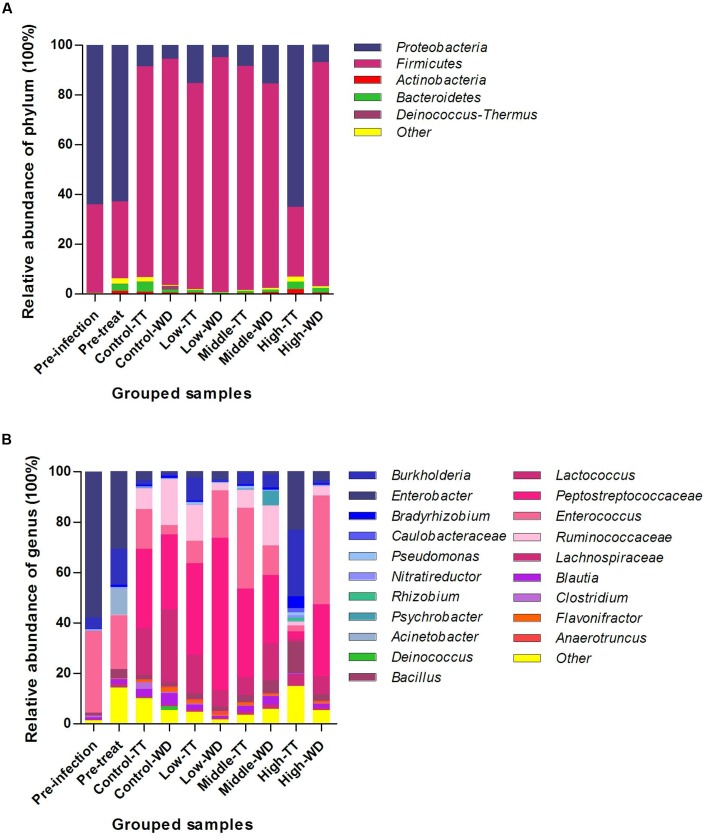
Taxonomic classification of the 16S rRNA sequences at phylum **(A)** and genus **(B)** levels. Data were pooled by treatment and dosages. TT, treatment; WD, withdrawal.

The results of phylum and genus distributions of microbial composition are shown in **Figure 4**. In total, 28 phyla were identified for the four groups. *Proteobacteria* was the most prevalent phylum at timepoints of pre-infection (63.93%) and pre-treatment (62.91%). For samples collected post treatment, *Firmicutes* was the most dominant phylum and made up 82.15–94.37% of the total population for most groups except for the treatment period of the high dosage group (**Figure 4A**). The high dosage group’s treatment time was dominated by *Proteobacteria* (64.95%), which was significantly higher than that in the other three groups and the withdrawal period in the same group (**Supplementary Figure S1A**). *Firmicutes* was concurrently significantly decreased (**Supplementary Figure S1B**). No significant differences were observed for these two phyla when comparing the withdrawal periods among the four groups. The other differentially abundant phyla among the four groups identified by Metastats (*p* < 0.05) were *Cyanobacteria*, *Deinococcus-Thermus*, *Planctomycetes*, *Chlorobi*, *Fibrobacteres*, *Acidobacteria* and *Fusobacteria*.

Of all the 480 different genera detected, 20 genera were defined as abundant genera (>1% of the total sequences) and made up 84.98–98.22% of the total population in the four groups (**Figure 4B**). For the time points of pre-infection and pre-treatment, *Enterobacter* was the most prevalent genus in Proteobacteria, followed by *Burkholderia*, and the Firmicutes was dominated by *Enterococcus*. For most of the grouped samples except the high dosage group’s treatments, the prevalent phylum Firmicutes was mainly comprised of *Peptostreptococcaceae* (27.26–60.46%), *Enterococcus* (3.87–43.12%), *Ruminococcaceae* (2.85–18.02%) and *Lachnospiraceae* (6.59–29.05%). In the treatment period of the high dosage group, *Burkholderia* (26.37%) was of highest abundance in the dominate phylum Proteobacteria.

All the differentially abundant genera (*n* = 113) identified by Metastats (*p* < 0.05) are illustrated in **Supplementary Figure S2**. The abundances of five genera (*Anaerotruncus*, *Blautia*, *Janibacter*, *Flavisolibacter*, and *Parasutterella*) were significantly decreased in the high dosage group than in the control group for both the treatment period and the withdrawal period, as shown in **Supplementary Figure S2**, bracket A. A total of 23 genera were significantly reduced in the medicated groups than in the control group and the differences were only observed in the treatment period (**Supplementary Figure S2**, bracket B). Abundances of seven genera (*Chthoniobacter*, *Quadrisphaera*, *Salinimicrobium*, *Haliscomenobacter*, *Clostridium*, *Aquicella*, and *Nitrosococcus*) were consistently declined in the three medicated groups than in the control group in the withdrawal period (**Supplementary Figure S2**, bracket C). Of all the 61 genera which were significantly different among the treatments of the four groups, 25 genera were significantly enriched in the high dosage group than in the control group, including both probiotics (e.g., *Bacillus* and *Lactococcus*) and recognized pathogens (e.g., *Burkholderia*, *Pseudomonas*, *Streptococcus*, *Klebsiella*, *Acinetobacter*) (**Supplementary Figure S2**, bracket D). The differentially abundant genera were also examined between the treatments and withdrawals within each group. Most of the significant differences were observed in high dosage group (38/64). Of all the 38 differentially abundant genera detected in the high dosage group, the relative abundances of 35 genera were significantly increased in the treatment period (**Supplementary Figure S2**, bracket E).

**FIGURE 5:**
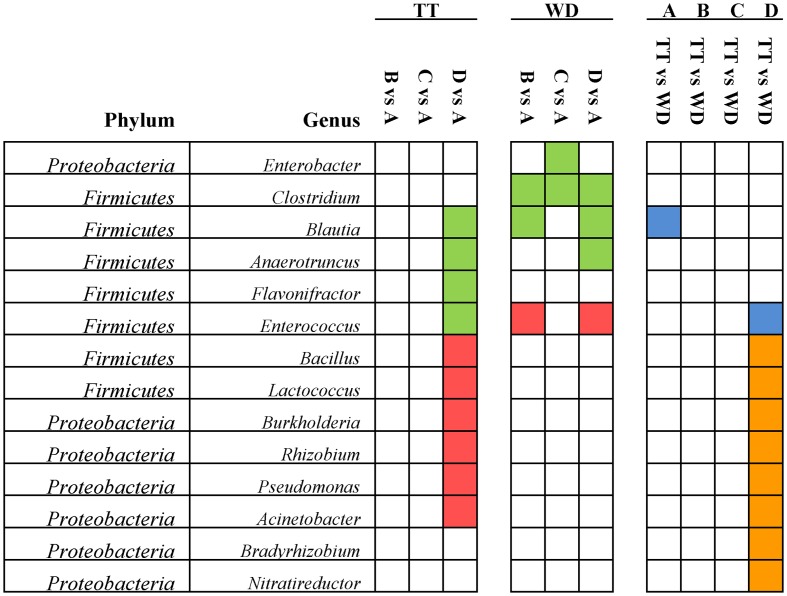
Significant differences of the abundances in the 14 abundant genera (>1% of the total sequences) among the four groups identified by Matastats (*p* < 0.05). In the comparisons of each medicated group with control group for the treatment and withdrawal period, respectively, genera whose abundances were higher in the medicated group than in the control group were marked with red color, otherwise marked with green. In the comparisons of treatment and withdrawal within each group, genera whose abundances were higher in the treatment period than in the withdrawal period were marked with orange color, otherwise marked with blue. TT, treatment; WD, withdrawal; A, control group; B, low dosage group; C, PK/PD dosage group; D, high dosage group.

Of all the 20 abundant genera (>1% of the total sequences), 14 genera presented significant differences and they were summarized in **Figure 5**. In Firmicutes, four abundant genera (*Blautia*, *Anaerotruncus*, *Enterococcus*, and *Flavonifractor*) were significantly reduced in the treatment period of the high dosage group, while *Bacillus* and *Lactococcus* were significantly increased, and in the withdrawal period the *Enterococcus* was significantly enriched. Four abundant genera (*Burkholderia*, *Rhizobium*, *Pseudomonas*, and *Acinetobacter*) from Proteobacteria were significantly increased, which might account for the increase of Proteobacteria in the treatment time of the high dosage group.

**FIGURE 6:**
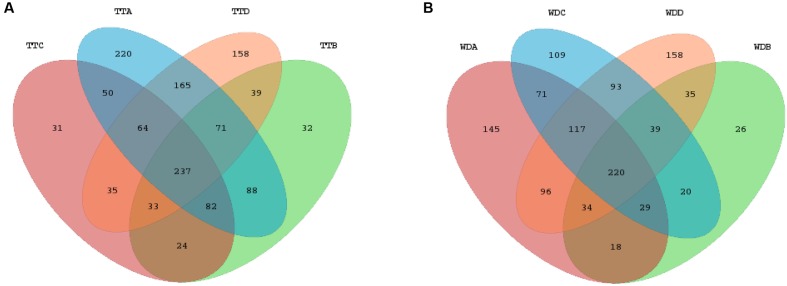
Venn diagrams of the core and unique OTUs of the four groups for the treatment **(A)** and withdrawal **(B)** period, respectively. TT, treatment; WD, withdrawal; A, control group; B, low dosage group; C, PK/PD dosage group; D, high dosage group.

Venn diagrams were generated to make qualitative comparisons of the core and unique OTUs between treatments and withdrawals in each group (**Supplementary Figures S3A–D**), among the treatments of the four groups (**Figure 6A**) and among the withdrawals of the four groups (**Figure 6B**). Representative sequences for each OTU were retrieved and classified to genus level. The 237 core OTUs of the four groups’ treatments were classified to 91 different genera and the 220 core OTUs of 78 genera were shared by the four groups’ withdrawal period. Of these two set of core genera, 63 genera were presented in both periods, including all the 14 differentially abundant genera. The unique OTUs were assigned to genus and compared among groups. In comparison of the four groups’ treatments, the numbers of unique genera for each group were 39 (control group), 2 (low dosage group), 6 (PK/PD dosage group) and 32 (high dosage group), respectively. The unique genera in the withdrawal periods of the four groups were 29 for control group, 3 for low dosage group, 29 for PK/PD group and 30 for high dosage group.

**FIGURE 7:**
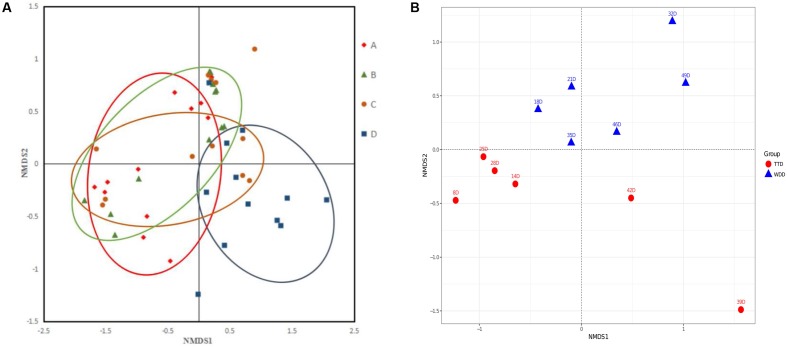
The non-metric multidimensional scaling (NMDS) plots of the samples in the four groups **(A)** and the samples in the treatments and withdrawals of the high dosage group **(B)**. A, control group; B, low dosage group; C, PK/PD dosage group; D, high dosage group. TT, treatment; WD, withdrawal.

The NMDS analysis was conducted to determine the changes of community structure of the four dosage groups. The microbial community of high dosage group was clearly separated from the other three groups (**Figure 7A**). Low dosage group and PK/PD dosage group were not clustered separately from the non-medicated group. The results of ANOSIM revealed that the microbial community structure changed among the four groups [R = 0.20, *p* = 0.003]. In the high dosage group, the individual samples of the treatments were clearly separated from the withdrawals (**Figure 7B**) and the analysis of the ANOSIM confirmed the significant changes [R = 0.35, *p* = 0.009]. However, the treatments and withdrawals samples were not clustered separately in the other three groups (**Supplementary Figures S4A–C**).

### Microbial Functions Prediction

**FIGURE 8:**
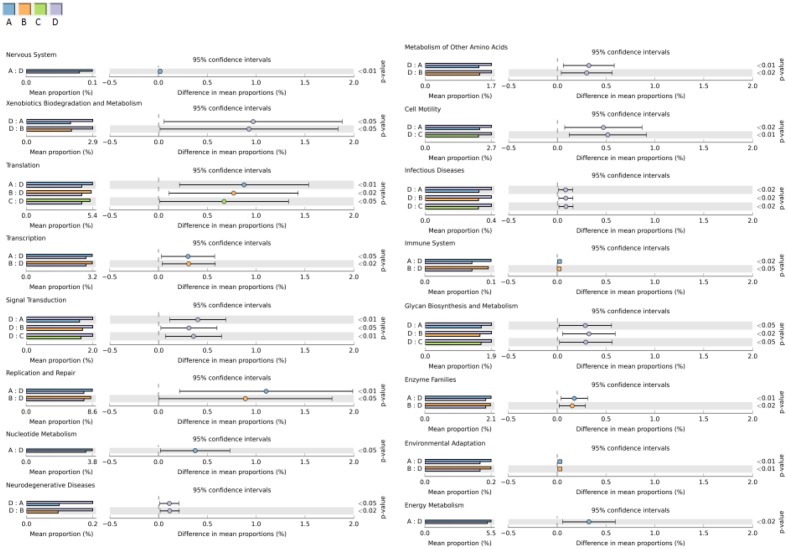
Comparisons of predicted functional pathways among microbes of the four groups. A, control group; B, low dosage group; C, PK/PD dosage group; D, high dosage group.

PICRUSt was used to predict the functional pathways of microbes in the four groups. Of the 41 subsystems observed, 16 subsystems was identified as significantly different among the four groups (*p* < 0.05) (**Figure 8**). The subsystems related to metabolism (xenobiotics biodegradation and metabolism), environmental information processing (signal transduction), infectious diseases and cellular processes (cell motility) were significantly enriched in the high dosage group than the other three groups. While the functional groups involved in genetic information processing (translation, replication and repair and transcription), organismal systems (environmental adaptation) and metabolism (nucleotide metabolism, enzyme families and energy metabolism) were shown to be more frequently detected in the control group and low dosage group.

## Discussion

Using the 16S rRNA gene sequencing approach, we have investigated for the first time the impact of repeated enrofloxacin treatments on the intestinal microbiota of *Salmonella* infected chickens.

Chicken gut microflora is a complex and diverse system, undergoes maturation from birth to adulthood (Choi et al., 2015) and it may be affected by many factors such as environment, age, feed additive, antibiotic, hygiene level, diet, breed, geography and climate (Qu et al., 2008; Danzeisen et al., 2011). In the present study, all the chickens were kept under same conditions and in different isolators with no possibility of contamination from external sources of microbiota. All birds were supplied with the same feed and water during the entire experiment. We believe that the differences of the gut microbiota composition among the four groups are direct consequences of the antibiotic therapy alone. Since the majority of microbes in chicken feces were same with the large intestine and could be repeatedly sampled from the same chicken for the longitudinal analysis of gut microbiota composition (Kim et al., 2012), we elected to choose feces as the collected samples in this study. Bacterial 16S rRNA genes contain nine “hypervariable regions” which could be used to classify bacteria (Van de Peer et al., 1996; Kim et al., 2011). V4 region has been chosen in this study for its high classification consistency (Ong et al., 2013; Zhao et al., 2013).

The average values of diversity were higher in the control group than in the three medicated groups (**Table 2**), indicating that enrofloxacin treatment seemed to reduce the bacterial species in the chickens gut. This finding is consistent with previous studies. Dethlefsen et al. (2008) reported that administration of ciprofloxacin to human affected the bacterial taxa in human gut and resulted in decreased richness and diversity of microbiota. The NMDS plot patterns and ANOSIM analysis indicated that enrofloxacin at a high dosage of 100 mg/kg b.w. significantly changed the microbial community structure in comparison with the other three groups (**Figure 7A**). The samples from the treatments and withdrawals of the high dosage group were also clearly separated (**Figure 7B**). Combing with the result of the microbiota composition, the changes of the microbial structure were mainly due to the increase of phylum *Proteobacteria* and concurrent decrease of *Firmicutes* in the treatment period (**Figure 4A**).

Several studies have revealed the gut microbiota composition in chickens using high throughput sequencing method (Yin et al., 2010; Danzeisen et al., 2011; Singh et al., 2012; Stanley et al., 2013; Sergeant et al., 2014; Mohd Shaufi et al., 2015). Due to the differences in sequencing techniques and concepts of the study, it may be inaccurate to directly compare the results of present study and previous reports. Based on our results, the fecal microbial communities were dominated by *Firmicutes* and *Proteobacteria*, which made up more than 90% of total sequences. A large proportion of the *Firmicutes* was classified to genera *Peptostreptococcaceae*, *Enterococcus*, *Ruminococcaceae* and *Lachnospiraceae*, which was in accordance with other reports in chicken (Lu et al., 2003; Jozefiak et al., 2010; Singh et al., 2012). The anatomical physiology and feeding habits of chickens (mainly fed with fiber and use the caecum and large colon for fermentation) may account for the predominance of *Firmicutes*, which are heavily comprised of anaerobes (Kim et al., 2011; Costa et al., 2012; Liu et al., 2014).

In total, 23 genera were significantly decreased (**Supplementary Figure S2**, bracket B) and 25 genera were significantly enriched in the medicated group than in the control group (**Supplementary Figure S2**, bracket D), however these differences were only observed in the treatment period with no such differences detected in the withdrawal period. Given that the chicken gut microbial community is a complex and relative stable ecosystem, the restoration of the microbiota after therapy withdrawal could be expected (Robinson et al., 2010; Macfarlane, 2014). However, the abundances of five genera (*Parasutterella*, *Flavisolibacter*, *Janibacter*, *Blautia*, and *Anaerotruncus*) were significantly decreased in the medicated groups than in the control group for both the treatment period and the withdrawal period (**Supplementary Figure S2**, bracket A). It seems that these genera of bacteria are highly susceptible to antibiotics and they might be eradicated by the high dosage of enrofloxacin with no recovery after therapy withdrawal. This finding was consistent to previous studies. In a study investigating the effects of oral florfenicol and azithromycin on gut microbiota in mice, the researchers also observed that the abundance of *Anaerotruncus* declined in the florfenicol group and *Parasutterella* decreased in both the antibiotic groups (Li et al., 2017).

Of the 25 significantly increased genera in the treatment period of the high dosage group, 6 genera were defined as the abundant genera (>1% of the total sequences), including two probiotics (*Lactococcus* and *Bacillus*), three recognized pathogens (*Burkholderia*, *Pseudomonas*, *Acinetobacter*) and *Rhizobium* (**Figure 5**). Two possible explanations are suggested to account for the significant enrichment of these genera under the pressure of enrofloxacin treatment. Firstly, several previous studies reported that *Lactococcus*, *Burkholderia* and *Bacillus* were associated with intrinsic ciprofloxacin resistance (Mathur and Singh, 2005; Fajardo et al., 2008; Walsh and Duffy, 2013; Huang et al., 2016). Secondly, resistant mutant might be selected under the antibiotic pressure and lead to the propagation. An *in vitro* study showed that mutants with high-level ciprofloxacin resistance were selected in *Pseudomonas aeruginosa* populations exposed to sub-MICs of ciprofloxacin (Jorgensen et al., 2013). The point mutations in target site genes contribute to the FQs-resistance in *Pseudomonas aeruginosa*, while impose a fitness cost which might account for the decrease in the withdrawal period (Agnello et al., 2016).

Some genera were significantly decreased in the medicated groups than in the control group, but this fact was only observed in the withdrawal period, with no significant differences detected in the treatment period (**Supplementary Figure S2**, bracket C). This trend may be due to the bacterial stress response reaction (Mazel and Mobashery, 2012). These genera of bacteria may survive under the antibiotic pressure by mounting responses in gene expression and protein activity. A variety of physiological processes of bacteria might be changed and the bacterial survivability and colonization ability may be decreased during the stress response, leading to the competitively exclusion by other commensal bacteria in the withdrawal period (Martinez and Rojo, 2011; Poole, 2012). These genera may be important potential targets for fluoroquinolones antimicrobial effects. Moreover, the fact of diminishing of taxa in response to antimicrobial therapy was also reported in other studies (Brandl et al., 2008; Dethlefsen et al., 2008; Hill et al., 2010; Looft and Allen, 2012). In a microbial community analysis of humans in response to ciprofloxacin, 4 weeks after the withdrawal of ciprofloxacin, the microbiota of the treated individuals merely resembled the pretreated state, and several taxa did not recover (Dethlefsen et al., 2008).

The potentially pathogenic bacterium *Clostridium* was significantly decreased in all the three medicated groups than in the control group for the withdrawal period (**Figure 5**). There are three species of *Clostridium* that can cause serious diseases: *Clostridium tetani* (tetanus), *Clostridium botulinum* (botulism) and *Clostridium perfringens* (gas gangrene). *Clostridium perfringens* has long been recognized as the etiological agent of the necrotic enteritis in chickens (Fasina et al., 2016). The reduction of *Clostridium* may represent a beneficial effect of enrofloxacin treatment regardless of dosages. Another interesting finding was that in the high dosage group, the genus *Enterococcus* was significantly reduced in the treatment period and significantly enriched after withdrawal of mediation (**Figure 5**). This finding was consistent to previous studies. Videnska et al. (2013) reported that *Enterococcus* in the chicken fecal microbiota was increased in response to the single or repeated therapy with tetracycline and streptomycin. Lower concentrations of ciprofloxacin in soil also enriched the genus *Enterococcus* (Huang et al., 2016) *Enterococcus* has shown to be intrinsically resistant to several antibiotics, such as β-lactams and aminoglycosides. The emergence of virulent *Enterococcus* that is resistant to vancomycin (VRE) has widely raised public concern. The increase of this opportunistic pathogen during the withdrawal period may be a result of resistance selection. A study by Brandl et al. (2008) showed that depletion of commensal microbes in response to broad-spectrum antibiotics can provide space and nutrients for pathogenic organisms, reduce the antimicrobial molecules in the intestinal mucosa, impair the mucosal innate immune defenses and lead to the increasing susceptibility to proliferated vancomycin-resistant *Enterococcus* (VRE) (Brandl et al., 2008). Results from the present study indicates that the enrichment of *Enterococcus* after enrofloxacin treatment should be highlighted and the susceptibility to antimicrobials needs to be investigated by further studies.

PICRUSt was used to predict the microbial functions and differences among the four groups and were analyzed by the STAMP software (**Figure 8**). High dosage of enrofloxacin treatment showed significant effects on various biosynthetic pathways in the chicken gut microbiota. Several metabolism pathways were elevated in this group, including xenobiotics biodegradation and biosynthesis of other secondary metabolites (penicillin and cephalosporin biosynthesis). Meanwhile, cellular processes (cell motility) and environmental information processing and signaling (membrane and intracellular structural molecules, pores ion channels and membrane transport by secretion system) were increased, indicating a higher capability of microbiota in host adhesion, infection and colonization. However, the functions associated with genetic information processing, such as replication and repair (DNA replication, homologous recombination, mismatch repair and nucleotide excision repair), transcription (transcription machinery), translation (RNA transport) and folding, sorting and degradation (proteasome) were consistently much less detected. This finding would be expected since the bactericidal activity of enrofloxacin is mainly mediated by targeting on DNA gyrase and DNA topoisomerase IV, which facilitate DNA replication, recombination, and transcription (Fabrega et al., 2009).

## Conclusion

This study showed that high dosage of enrofloxacin (100 mg/kg b.w.) was effective in eradicating *Salmonella* from chickens gut. However, the microbial community membership and structure, and microbial functions were strongly affected during the treatment. Several genera were significantly decreased, which might be the potential targets for the fluoroquinolones antimicrobial effects. This study provides a more comprehensive glimpse at the chickens gut microbial modulations in response to different dosages of enrofloxacin and will aid in the prudent and rational use of antibiotic in poultry industry.

## Author Contributions

JL performed experiments, analyzed the data and wrote manuscript; HhH, MD, and GC designed the experiments, analyzed the data and edited the manuscript; CL, SA, MS, and HfH performed experiments; ZY supervised and coordinated the whole project.

## Conflict of Interest Statement

The authors declare that the research was conducted in the absence of any commercial or financial relationships that could be construed as a potential conflict of interest.

## Supplementary Material

**FIGURE S1 |** Significant differences of relative abundances of phylum *Proteobacteria* **(A)** and *Firmicutes* **(B)** between the treatments and withdrawals of the four groups. TT, treatment; WD, withdrawal.

**FIGURE S2 |** Differentially abundant genera identified by Matastats (*p* < 0.05). In the comparisons of each medicated group with control group for the treatment and withdrawal period, respectively, genera whose abundances were higher in the medicated group than in the control group were marked with red color, otherwise marked with green. In the comparisons of treatment with withdrawal in each group, genera whose abundances were higher in the treatment period than in the withdrawal period were marked with orange color, otherwise marked with blue. TT, treatment; WD, withdrawal; A, control group; B, low dosage group; C, PK/PD dosage group; D, high dosage group.

**FIGURE S3 |** Venn diagram of the core and unique OTUs of the treatment **(A)** and withdrawal **(B)** period in each group. TT, treatment; WD, withdrawal; **(A)** control group; **(B)** low dosage group; **(C)** PK/PD dosage group; **(D)** high dosage group.

**FIGURE S4 |** The non-metric multidimensional scaling (NMDS) plots of the treatments and withdrawal in each group. TT, treatment; WD, withdrawal; **(A)** control group; **(B)** low dosage group; **(C)** PK/PD dosage group.
