# Supplementary figures and images for: Microbial Shifts in the Intestinal Microbiota of Salmonella Infected Chickens in Response to Enrofloxacin
Source: Front Microbiol. 2017 Sep 8;8:1711. doi: 10.3389/fmicb.2017.01711 (PMC5596078; doi:10.3389/fmicb.2017.01711)

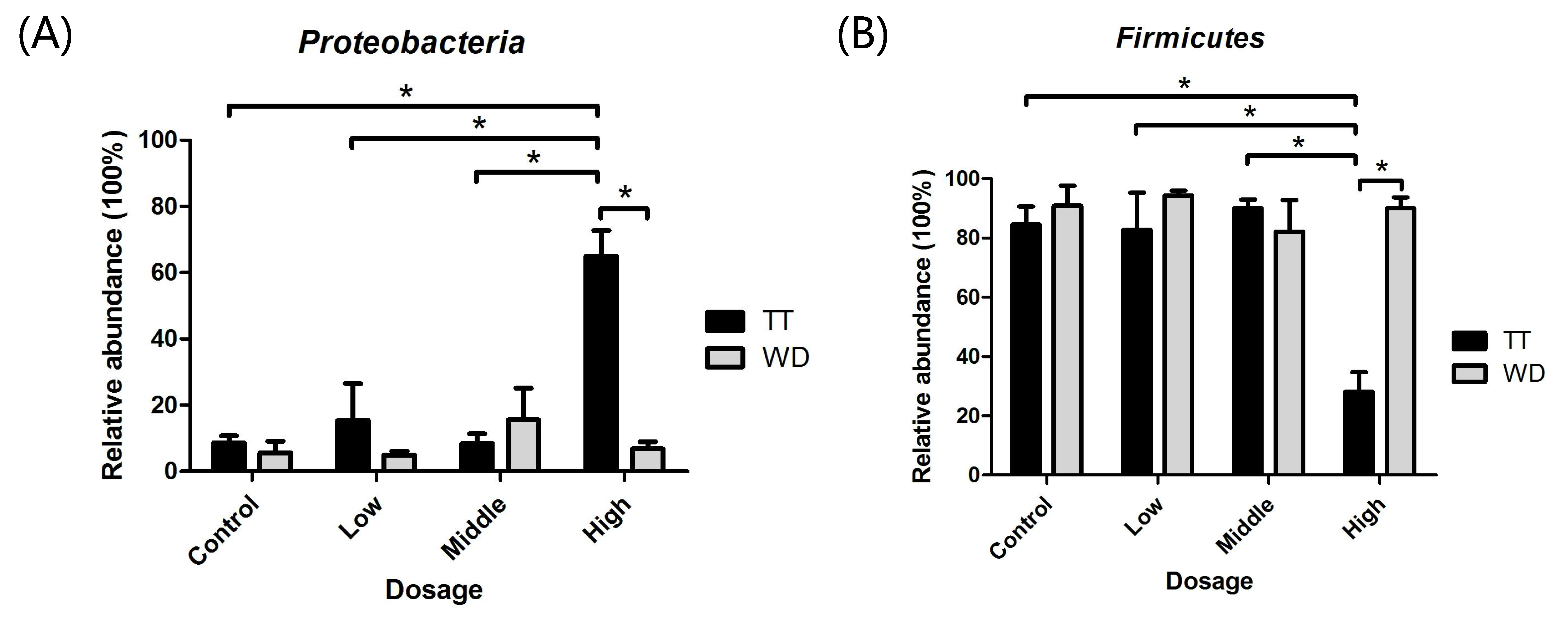

Supplement: Supplementary file 1 [file Image_1.TIFF]

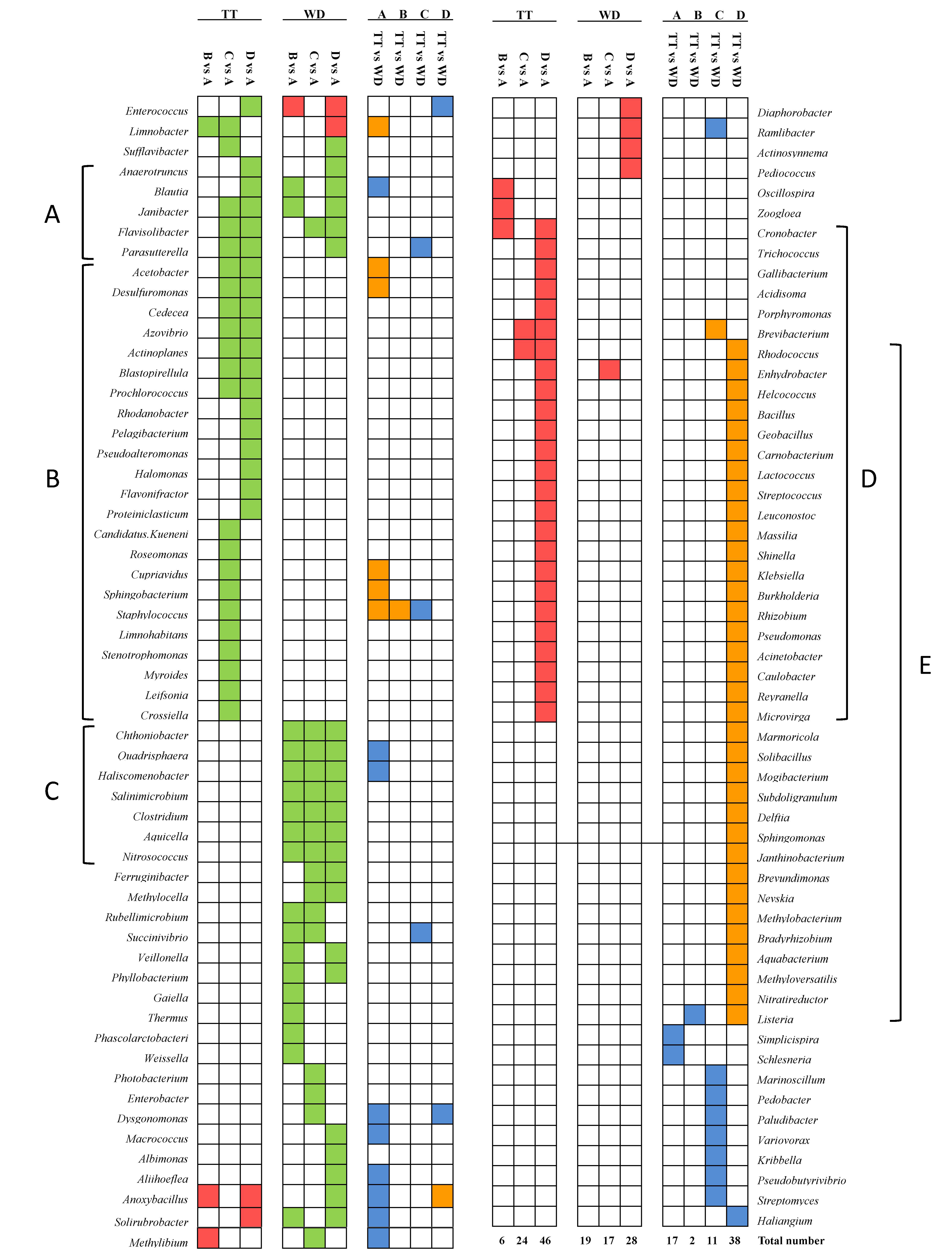

Supplement: Supplementary file 2 [file Image_2.TIFF]

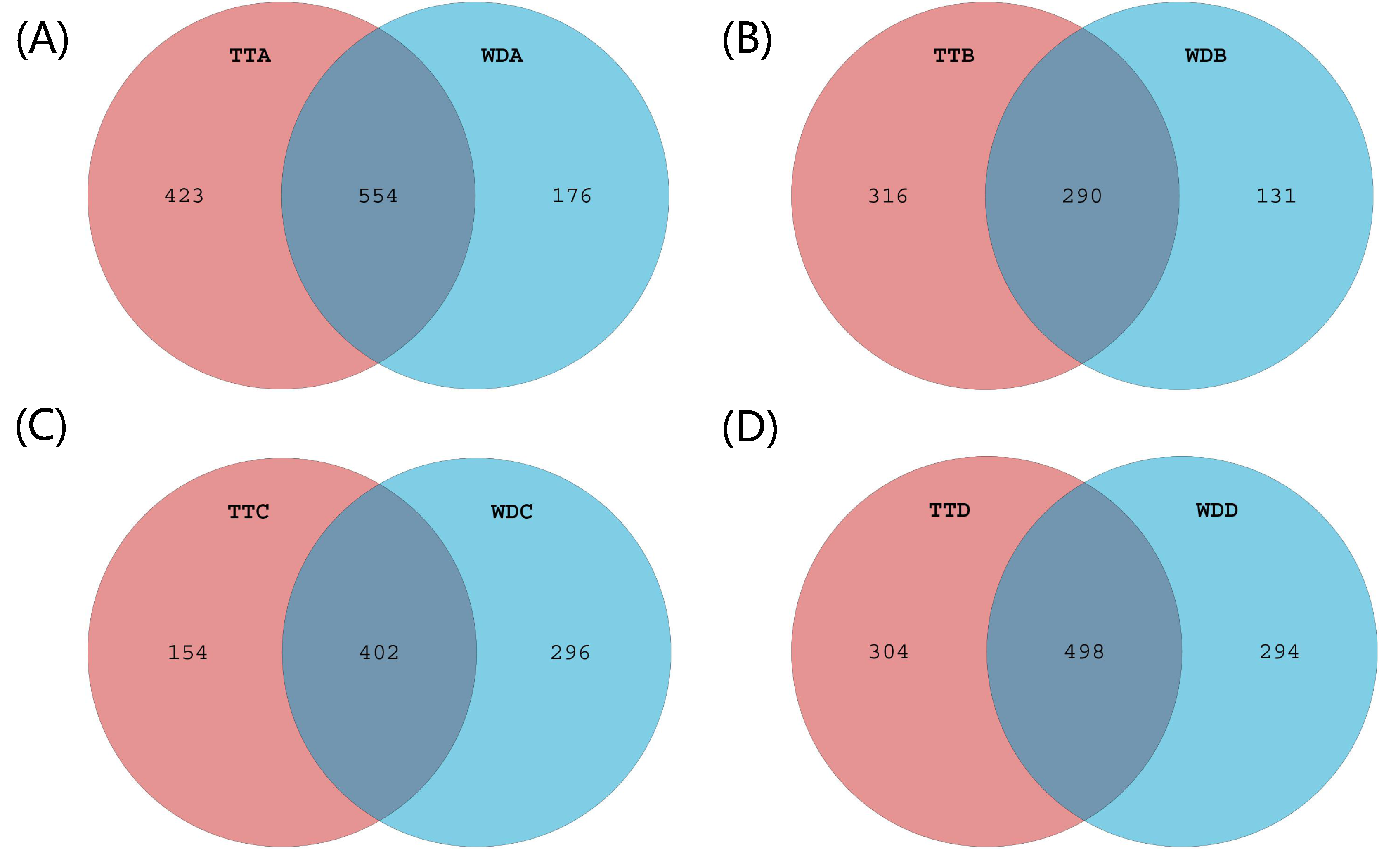

Supplement: Supplementary file 3 [file Image_3.TIFF]

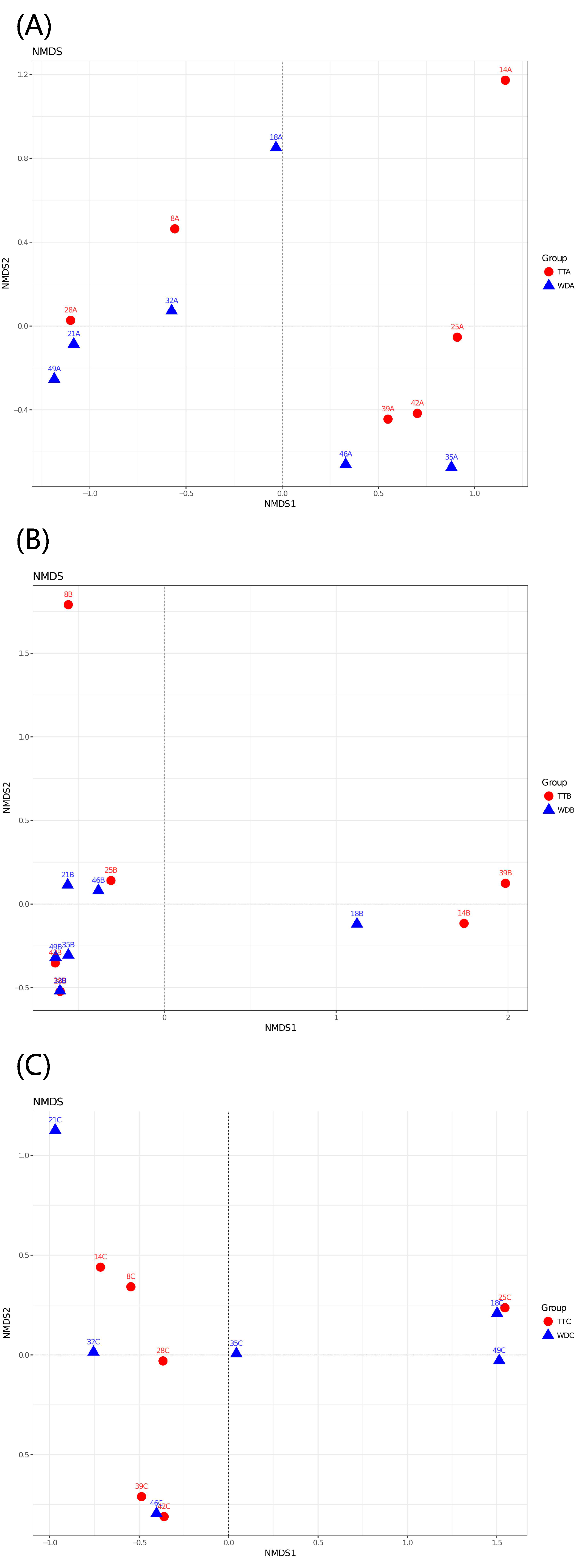

Supplement: Supplementary file 4 [file Image_4.TIFF]
